# Connectivity of the Human Number Form Area Reveals Development of a Cortical Network for Mathematics

**DOI:** 10.3389/fnhum.2018.00465

**Published:** 2018-11-26

**Authors:** Federico Nemmi, Margot A. Schel, Torkel Klingberg

**Affiliations:** ^1^Department of Neuroscience, Karolinska Institute, Stockholm, Sweden; ^2^INSERM U1214 Centre d’Imagerie Neuro Toulouse, Toulouse, France; ^3^Institute of Psychology, Leiden University, Leiden, Netherlands

**Keywords:** mathematics development, mathematics brain network, functional connectivity development, category specific brain areas, visual word form area

## Abstract

The adult brain contains cortical areas thought to be specialized for the analysis of numbers (the putative number form area, NFA) and letters (the visual word form area, VWFA). Although functional development of the VWFA has been investigated, it is largely unknown when and how the NFA becomes specialized and connected to the rest of the brain. One hypothesis is that NFA and VWFA derive their special functions through differential connectivity, but the development of this differential connectivity has not been shown. Here, we mapped the resting state connectivity of NFA and VWFA to the rest of the brain in a large sample (*n* = 437) of individuals (age 3.2–21 years). We show that within NFA-math network and within VWFA-reading network the strength of connectivity increases with age. The right NFA is significantly connected to the right intraparietal cortex already at the earliest age tested (age 3), before formal mathematical education has begun. This connection might support or enable an early understanding of magnitude or numerosity In contrast, the functional connectivity from NFA to the left anterior intraparietal cortex and to the right dorsolateral prefrontal cortex is not different from the functional connectivity of VWFA to these regions until around 12–14 years of age. The increase in connectivity to these regions was associated with a gradual increase in mathematical ability in an independent sample. In contrast, VWFA connects significantly to Broca’s region around age 6, and this connectivity is correlated with reading ability. These results show how the differential connectivity of the networks for mathematics and reading slowly emerges through years of training and education.

## Introduction

Over the last decades several fMRI studies have investigated the functional organization of the occipito-temporal cortex in humans. Several brain regions in the occipito-temporal cortex have been defined by selectivity for object shape, or by selectivity for specific categories, such as bodies (fusiform body areas, Schwarzlose et al., 2005), faces (fusiform face area, Kanwisher et al., 1997), and places (parahippocampal place area, Epstein and Kanwisher, 1998).

Surprisingly, specificity in the occipitotemporal lobe has been found also for evolutionary recent stimuli. The visual word form area (VWFA) is an area in the left occipito-temporal cortex that consistently responds more to letters and words than to other stimuli (Cohen et al., 2000, 2002; Dehaene et al., 2002). The tuning of VWFA to its preferred stimuli seems to be a prolonged process, with response to words stabilizing at around 12 years of age (Ben-Shachar et al., 2011). Although there is no consensus about the role of this area in word-reading, it must be said that its specificity has been debated (Price and Devlin, 2003).

The VWFA is connected to temporo-parietal and inferior frontal (Broca’s area) language related regions (Bouhali et al., 2014). The connections between language related areas are present early, even before the beginning of literacy (Dehaene-Lambertz et al., 2006; Perani et al., 2011), and connectivity to VWFA from the temporal cortex is present before specific functional response to words (Saygin et al., 2016). However, the microstructural properties of VWFA anatomical connectivity is influenced by education, as subjects who have learnt to read in adulthood have higher fractional anisotropy in the arcuate fasciculus than illiterate adults of the same age (Thiebaut de Schotten et al., 2014). One study (Vogel et al., 2012) failed to find functional connectivity between VWFA and reading regions, but found the VWFA to be functionally connected to regions belonging to the dorsal attentional network.

Less is known about the development of the recently discovered area specialized for symbolic numbers representation: the putative number form area (NFA). Selective response to symbolic numbers was first shown using electrophysiological recordings from the surface of the cortex (ECoG) from the right occipito-temporal cortex, in a position more anterior and lateral to VWFA (albeit on the right side) (Shum et al., 2013). More recently, the same region has been found bilaterally using fMRI in blind subjects (Abboud et al., 2015), in sighted subjects (Amalric and Dehaene, 2016; Grotheer et al., 2016b) and in expert mathematicians (Amalric and Dehaene, 2016). The fact that this region has been found in both sighted and non-sighted subjects suggest that even if it is specific for symbolic number representation, it is independent from sensory modality (Abboud et al., 2015). In the blind sample, this region has been found to be functionally connected to the right intraparietal sulcus (IPS) (Abboud et al., 2015), a region important for mathematics (Piazza et al., 2004; Castelli et al., 2006; Arsalidou and Taylor, 2011). The functional connectivity between NFA and IPS has been confirmed using ECoG (Daitch et al., 2016). Recently, a functional metanalysis showed that it was possible to observe a consistent cluster of activation related to Arabic numerals only when stringent criteria for contrasts inclusion were set (Yeo et al., 2017). Specifically, the contrasts included in the metanalysis that led to a significant cluster in the right inferior frontal gyrus (IFG) had to be matched for task demand (e.g., single digit compared to single letter) casting some doubts about the functional specificity of this region (Yeo et al., 2017). However, a recent ECoG study found that not only NFA responded more to numerals than number-words and words, it also shows greater activity when the subjects solved equations relative to when they have to remember sentences. Moreover, when equation where presented one digit at a time (e.g., 2 + 3 = 5) the activity associated with the second and third digits was higher than the activity associated with the first one (Hermes et al., 2017). This effect was present both when using digits and number-words. Taken together the results of Hermes et al. (2017) suggest that NFA is specific for numerals for passive viewing, but its activity increases when numerals (and number-words) are in an algebraic context. Hermes et al. (2017) suggested that the task-modulation they found in NFA was caused by its connection with other areas related to mathematical cognition (e.g., the IPS), thus making this region interesting beyond its specific role in numerals processing. Task-related modulation of responses has been reported in the VWFAs (Song et al., 2012).

Despite the growing evidences of number specific processing in the right inferior temporal lobe, little is known about the development of the connectivity of this area during childhood and adolescence (Hannagan et al., 2015). It has been proposed that NFA and VWFA derive their specificity from their pattern of connectivity. VWFA could be specialized for processing letters because of its connection with language areas, while NFA is specific for symbolic numbers because of its connection with the IPS (Hannagan et al., 2015). However, little is known about the specific functional connectivity profile of VWFA and NFA, how it changes during development, and its relation to performance.

In this paper, we selected the NFA ROI based on a previous study that included adults (Grotheer et al., 2016b) and we analyzed how this putative-NFA network develop from childhood into adulthood using resting state functional connectivity. We hypothesized that through education and development, NFA connectivity would change, showing a strengthening in key regions. This strengthening would in turn be related to improvement in mathematical performance during development. Alternatively, a specific functional connectivity could be established very early in life, before formal education, paralleling anatomical connectivity (Saygin et al., 2016). These two mechanisms could both be at play, with some regions in the network showing connectivity early in life and other regions increasing their connectivity with NFA during development.

As a comparison, we observed development of VWFA functional connectivity, whose connectivity and development are better known (Dehaene-Lambertz et al., 2006; Perani et al., 2011; Dehaene et al., 2015). VWFA is the best control region for NFA, as they share a similar anatomical location and a similar functional profile. As a further control, and to exclude that results observed for NFA and VWFA could be related to spurious factors, we also observed the development of the connectivity in the sensorimotor network: since the somatosensory and motor areas develop early in life we expected significant connectivity within these regions, but no effect of age on connectivity.

To test our hypothesis, we analyzed resting state functional connectivity (rs-FC) data from two independent developmental samples. We aimed to investigate the existence of a differential connectivity profile of NFA and VWFA. Then we analyzed age-related increases in connectivity within NFA-mathematical and VWFA-reading networks.

Finally, we analyzed the association between the increase in connectivity within these networks, and developmental improvements in reading and mathematical performance.

## Materials and Methods

### Pediatric, Imaging, Neurocognition and Genetics (PING)

Data used in the preparation of this article were obtained from the Pediatric Imaging, Neurocognition and Genetics (PING) Study database^1^. The primary goal of PING has been to create a data resource of highly standardized and carefully curated magnetic resonance imaging (MRI) data, comprehensive genotyping data, and developmental and neuropsychological assessments for a large cohort of developing children aged 3–20 years. The scientific aim of the project is, by openly sharing these data, to amplify the power and productivity of investigations of healthy and disordered development in children, and to increase understanding of the origins of variation in neurobehavioral phenotypes. For up-to-date information, see http://ping.chd.ucsd.edu/.

The PING dataset is a multimodal imaging sample that is the result of a collaboration of several American universities^2^. Importantly for this study, PING includes subjects in preschool and in the first years of primary school (i.e., subjects who have not been exposed or have just started to be exposed to formal reading and mathematical teaching).

From the PING sample we included 437 subjects (mean age = 14.2 ± 4.7 years, range 3.2–21, 209 males) that had usable rs-fMRI and T1 images. Subjects were scanned using GE, Siemens and Philips scanners. The acquisition parameters were standardized within scanner and were as follows: for the Siemens scanners, rs-fMRI volumes were acquired using an EPI sequence with TR = 3000 ms, TE = 30 ms, in plane resolution = 3 mm × 3 mm, slice thickness = 3.5 mm, structural volumes were acquired using an MPRAGE sequence with TR = 2170 ms, TE = 4.33 ms, 160 sagittal slice, 1 mm^3^ isotropic resolution; for the Philips scanner, rs-fMRI volumes were acquired using an EPI sequence with TR = 2500 ms, TE = 30 ms, in plane resolution = 2.67 mm × 2.67 mm, slice thickness = 3 mm, structural volumes were acquired using an MPRAGE sequence with TR = 6.8 ms, TE = 3.1, 170 sagittal slices, 1 mm × 1 mm × 1.2 mm voxel; for the GE scanner, rs-fMRI volumes were acquired using an EPI sequence with TR = 3000 ms, TE = 30 ms, in plane resolution = 3 mm × 3 mm, slice thickness = 3 mm. For all scanner manufacturers the resting state acquisition consisted of 200 volumes. Subjects were instructed to rest still in the scanner while fixating on a white cross on black background.

#### ROI Selection and Analyses

**FIGURE 1:**
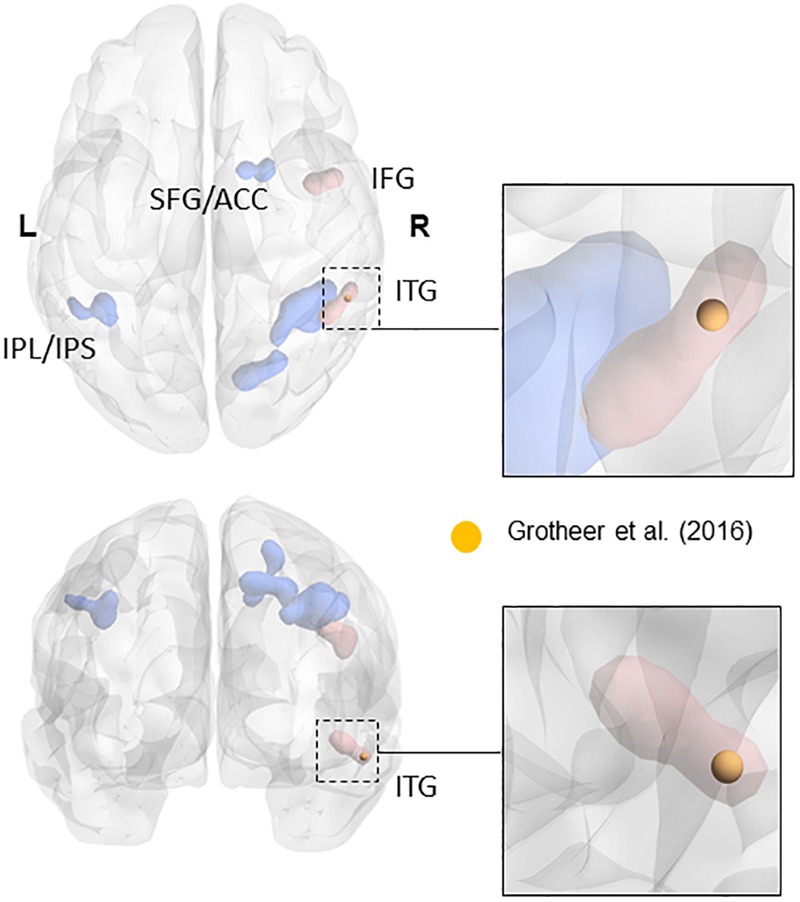
Overlap of the NFA-ROI used in the current paper (yellow) based on Grotheer et al. (2016b), with the meta-analytical clusters of math-related activity. Clusters that were present in the metanalysis both when using all contrasts and when using only contrasts matched for task demand (e.g., single digits compared to single characters) are in blue. Clusters that only appeared when using contrasts matched for task-demanded are in red (see Yeo et al., 2017 for more details). The insets show the cluster within the inferior temporal gyrus (in red). Adapted from a picture courtesy of Yeo et al. (2017).

The ROIs for NFA and VWFA were selected from prior literature. To our knowledge, only three papers have located NFA using fMRI (Abboud et al., 2015; Amalric and Dehaene, 2016; Grotheer et al., 2016b). Since the sample in the work by Abboud et al. (2015) comprised of blind people and the paper from Amalric and Dehaene (2016) was not focused on NFA, we chose to use the peaks in the study by Grotheer et al. (2016b) as ROIs, in which both NFA and VWFA were observed. In particular, we used the peak for the contrast numbers versus other stimuli as the center of gravity for our NFA ROI (*x* = 61, *y* = -45, *z* = -17), and the peak for the contrast letters versus other stimuli as center of gravity of our VWFA ROI (*x* = -46, *y* = -59, *z* = -15). All coordinates were in MNI space. The peak found in the study by Grotheer et al. (2016a), and used in the present study, falls within the metanalytic cluster found by Yeo et al. (2017) in their recent metanalysis (see Figure 1). Although we have no data to claim a functional specificity of this region for number in the two samples we used, the fact that our ROI fall within the metanalytical cluster found by Yeo et al. (2017) strongly suggest that we are indeed sampling signal from the area that has been labeled as the putative NFA. It is important to keep in mind that the study by Grotheer et al. (2016b) was performed within a sample of healthy *adults*. We are not discarding the possibility that in children (especially in the younger tail of the sample) VWFA and NFA differ in size and partially in location. However, the main hypothesis of this paper is that VWFA and NFA derive their functional specificity through change in functional connectivity occurring during development. As such using adult-based ROIs and observing their functional connectivity pattern during development seems a reasonable approach to test this hypothesis. Table 1 reports the coordinates of the peak labeled as NFA in the studies that have observed it, together with the distance from the center of gravity of our ROI.

**Table 1:** Peaks coordinates (MNI) of the NFA as observed in different ECoG (italic) and fMRI (plain) studies together with the distance from the center of gravity of our ROI (corresponding to the peak in Grotheer et al., 2016b).

	MNI coordinates of the peak (*x, y, z*)	Distance from the COG of our ROI (mm)
Abboud et al., 2015	55, -43, -20	7
Amalric and Dehaene, 2016	62, -39, -17	6
Grotheer et al., 2016a	61, -48, -13	5
Grotheer et al., 2016b	61, -45, -17	-
*Hermes* et al., *2017*	57, -51, -17	7
*Shum* et al., *2013*	51,- 54, -24	15
Yeo et al., 2017	47, -51, -21	16

*Note that the coordinates reported for Grotheer et al. (2016a) are the average coordinates of the peak observed at the single subjects level.*

We chose to mainly focus on the right NFA in this paper, as it is still debated if the NFA really is bilateral and Grotheer et al. (2016a) only showed functional relevance for NFA in the right hemisphere using TMS. Moreover, Hermes et al. (2017) showed an increase in the right hemisphere NFA response to numbers when presented in arithmetical context. Moreover, Yeo et al. (2017) only found metanalytic evidences for an NFA cluster in the right inferior temporal gyrus (ITG). However, as a control analysis, we also investigated the connectivity of a right-to-left flipped NFA ROI. The ROIs were created using Marsbar (Brett et al., 2002), with a radius of 6 mm around the center of gravity. A relatively small radius was chosen in order to assure that the ROIs would fall within the specific regions selected.

#### Resting State fMRI Preprocessing and Analysis

The rs-fMRI images were preprocessed and analyzed using CONN^3^ (Whitfield-Gabrieli and Nieto-Castanon, 2012). The volumes were slice timing corrected, realigned, normalized to the standard MNI T1 template via T1-coregistration and smoothed using a Gaussian kernel of 4 mm FWHM. The toolbox Artifact Detection Tools (ART)^4^ was used to identify volumes corrupted by excessive motion (defined as a frame-wise displacement > 2 mm or a root mean squared change in bold signal > 9). T1 images were segmented in gray matter, white matter and CSF using unified segmentation in SPM8. The time series was denoized using a previously described approach (Behzadi et al., 2007; Whitfield-Gabrieli and Nieto-Castanon, 2012): the time series extracted from the white matter and the CSF underwent a principal component analysis and the first five components from each time series were retained. These 10 components were entered in a linear model as variables, together with the six realignment parameters and, where relevant, the parameters from the ART toolbox coding for corrupted volume (Behzadi et al., 2007). The dependent variable of this model was the time series at each voxel within the gray matter. Subsequent analyses were performed using the residuals of this model, that is, the BOLD time series from each voxel in the gray matter, with the variance explained by white matter and CSF time series as well as movement removed. After regressing out the nuisance variables the time series were band-pass filtered between 0.008 and 0.09 Hz.

In order to account for the signal drop out usually observed in BOLD acquisition around the ventral aspect of the temporal lobe, we used the method proposed by Abboud et al. (2015) and formally tested in the study by Peer et al. (2016). Briefly, this method proposes to model the intensity of a BOLD image as a combination of two Gaussian distributions: one distribution modeling the voxels with low intensity (no brain or signal drop out voxels) and the other representing high intensity voxels. A threshold is set so that 99% of the low intensity voxels are excluded, and a binary mask created using this threshold. All subsequent analyses are performed on volumes that have been masked with this procedure. Although the metanalysis by Yeo et al. (2017) suggests that signal drop out is not the main factor driving the negative results about NFA, the masked procedure we used has been shown to generally increase the quality of resting state data (Peer et al., 2016).

We excluded all subjects that had more than 20% of the functional volumes censored due to movement artifacts or a total number of volumes after censoring lower than 80 (note that only two subjects had a number of volumes between 80 and 100, all other subjects had more than 115 volumes).

As a first step we performed two seed-to-voxel connectivity analyses, using VWFA and NFA as seeds, respectively. CONN calculates the correlation between the time series of the seeds (averaged among voxels within the seed) and the time series of each of the voxels in the brain. The correlation is then z-transformed using the Fisher transformation and significance is calculated. A threshold of *p* < 0.05 FDR corrected (Chumbley et al., 2010) was used as the statistical threshold for all the analyses.

As a second step, we contrasted the seed-to-voxel connectivity of VWFA and NFA. We performed two contrasts: VWFA > NFA and NFA > VWFA. In other words, we observed the voxels in the brain that were significantly more connected to VWFA than to NFA and vice versa.

In addition, we also tested for positive association between age and voxel-wise connectivity with VWFA and NFA (i.e., we observed those voxels in which the correlation between VWFA or NFA connectivity and age was significantly different from 0). We tested three different shape for the association between age and connectivity: a linear model (connectivity = C = β1 + β2^∗^age), an hyperbolic model with steep increase in the younger subjects and a plateau at older age (C = β1 – β2/age), and a second order model (inverted u-shaped, C = β1 + β2^∗^age – β3 ^∗^ age^2^). All the contrasts included acquisition protocol as confound variable.

### BrainChild Sample

This sample included 61 subjects (mean age = 16.76 ± 5.2 years, range 7.9–28.9, 33 males) with usable rs-fMRI and structural acquisition as well as reading and mathematical proficiency measures.

While the PING dataset provided us with the statistical power necessary to explore the connectivity of VWFA and NFA, it lacked behavioral measures of reading and mathematics that would allow us to test for a functional meaning of the increase in connectivity. For this reason, we turned to a previously locally acquired developmental sample, extensively described elsewhere (Soderqvist et al., 2010; Dumontheil et al., 2011; Darki and Klingberg, 2015). Briefly, this is a sample of children and young adults from 5 to 28 years old that underwent an extensive behavioral assessment and an MRI protocol including rs-fMRI and a structural acquisition.

#### Behavioral Assessment

##### Mathematical abilities

The assessment of mathematical abilities was based on the Trends in Mathematics and Science Study (Martin et al., 2004) and the Basic Number Screening Test (Gillham and Hesse, 2001). There were four school-grade-dependent versions (grades 2, 4, 6, and 8, the latter test suitable for 14 years and older) each taking 30 min. The different versions had overlapping items, allowing us to later link the results. Versions for grades 2 and 4 assessed magnitude judgments, number sequence, and elementary arithmetic (i.e., addition, subtraction, division, multiplication, and fractions). Versions for grades 6 and 8 assessed elementary arithmetic and elementary algebra (i.e., simple equations with variables).

##### Reading comprehension

The assessment of reading comprehension was based on narrative and expository texts from the Progress in International Reading Literacy Study (Martin et al., 2003), and the International Association for the Evaluation of Educational Achievement Reading Literacy Study (Gustafsson and Rosen, 2004). As with the test of mathematical abilities, there were four school-grade-dependent versions with overlapping items (grades 2, 4, 6, and 8, the latter suitable for 14 years and older), each taking 30 min.

##### Behavioral data analysis

The raw mathematical scores were transformed to ability scores using the Rasch model (Bergman Nutley et al., 2010). The ability scores take into account the difficulty of the item and the ability of the participant, allowing for the combined analyses of different age groups, even though age groups did not perform the exact same tests, since these were age dependent (see Supplementary Materials for further details).

#### MRI Acquisition Parameter and Analyses

The MRI acquisitions were performed on a 1.5-T Siemens scanner (Siemens, Erlangen, Germany) equipped with an 8-channels coil. One hundred and fifty T2^∗^-weighted functional volumes were acquired using a gradient-echo-planar imaging (EPI) sequence (TR = 2500 ms, TE = 50 ms, in plane resolution = 3.4 mm × 3.4 mm, slice thickness = 4.4) while the subjects were instructed to keep their eyes closed and not to fall asleep. The resting-state sequence comprised 200 volumes. Structural volumes were acquired using a T1-weighted spin echo scan using a 3-dimensional MPRAGE sequence with TR = 2300 ms, TE = 2.92 ms, 176 sagittal slices, and 1 mm^3^ isotropic voxel size.

The rs-fMRI images were preprocessed and analyzed using the same pipeline used in the PING dataset, including the intensity thresholding. In this sample, we extracted the connectivity between VWFA and the clusters showing an age-related connectivity increase with VWFA, as well as the connectivity between NFA and the clusters showing an age-related connectivity with NFA in the PING dataset. We tested the association between the pairwise connectivity of VWFA-age related clusters with reading as well as the association between the pairwise connectivity of NFA-age related clusters with mathematical (limited to those clusters that were found in the parietal and frontal lobe, and excluding clusters that did not show seed-specific age increase, see the section “Results”).

As a control analyses, we have observed the connectivity of the sensorimotor network: in order to do so we have drawn four spherical ROIs with the same radius as NFA and VWFA (i.e., 6 mm) centered on the center of gravity of the left and right Postcentral Gyrus (lPoCG, rPoCG) and the left and right Precentral Gyrus (lPrCG, rPrCG) as defined by the aal atlas. We then measured the connectivity between rPoCG and the other three regions and the correlation of these connectivities with age.

#### Experimental Design and Statistical Analyses

##### Analyses in the PING dataset

The differential connectivity between NFA and VWFA in the PING dataset was tested using a massive univariate approach, fitting one mixed linear model for each voxel with connectivity between seed and voxel as dependent variable, one between factor “acquisition protocol” and one within factor “Seed” (i.e., NFA or VWFA). The relationship with age was investigated using a similar approach: a linear model was fitted for each voxel with connectivity between seed and voxel as dependent variable and “acquisition protocol” and “age” as between factor. We decided to focus on positive association between connectivity and age as we were interested in integration within the NFA and VWFA networks with age, and integration is better characterized as increase rather than decrease in connectivity.

The analyses for confirming the possible differential slope of the increase of connectivity with age (i.e., if the clusters found in the previous analysis showed a steeper increase in connectivity specifically for NFA or VWFA) was performed by means of a set of seven mixed linear models (one per cluster found, see results) with between factor age, within factor seed and a random intercept for each subject to account for repeated measures.

The analysis testing the age of differentiation for connectivity with NFA/VWFA (see the section “Results”) was performed fitting a linear model to the difference in connectivity between each cluster and NFA/VWFA and observing the age at which the lower confidence interval was higher than 0 (i.e., the fitting was significant different from 0). We used bootstrapping to obtain confidence interval around the estimation of the age of differentiation: we resampled with replacement the original sample (with the size of the sample size N’ of the resampled sample equal to the sample size N of the original sample) 5,000 times, each time fitting a linear model to the difference in connectivity between each cluster and NFA/VWFA and observing the age at which the lower confidence interval was higher than 0, thus obtaining a sample of 5,000 ages of differentiation. These values were used to calculate the confidence interval of the age of differentiation.

The control analyses performed within the somatosensory network included a set of three linear models (one for each presented couple of ROIs) with the connectivity between ROIs as dependent variable and age as independent variable.

##### Analyses in the BrainChild dataset

The association between age-related increase in connectivity in the NFA and VWFA networks and behavioral measures was tested using a set of linear models with the connectivity between the age-related clusters (see the section “Results”) and NFA/VWFA as dependent variable and the behavioral measures of reading and math performance as independent variables. The results of this analysis were corrected for multiple comparisons using FDR. The differential connectivity between NFA and VWFA in this sample was tested using the same analytical strategy as in the PING dataset.

For voxel-wise analyses all results were corrected using topographical FDR (Chumbley et al., 2010). Briefly, this procedure is an amelioration of the classical voxel-based FDR correction that takes into account the spatial structure of the signal, giving more sensitive results than classic FWE correction technique and more accurate spatial results than voxel-wise FDR technique (Chumbley et al., 2010).

## Results

### Identifying a Mathematical Network by Analysis of Differential Connectivity

In order to identify a network of cortical regions specifically connected to NFA, we placed a seed in a cluster identified as the right NFA (*x* = 61; *y* = -45; *z* = -17) in a previously published results (Grotheer et al., 2016b). The right NFA, rather than the left, was used based on the evidence that the right NFA shows a stronger response to numbers than the left (Shum et al., 2013; Grotheer et al., 2016a), as well as because in a recent meta-analysis, only the right NFA was observed. Next, we identified all voxels in the brain that were significantly more correlated with NFA than with VWFA [*x* = -46, *y* = -59, *z* = -15, coordinates based on the same paper (Grotheer et al., 2016b)]. By contrasting the connectivity of NFA and VWFA we identified regions that were specific for NFA and subtracted away possible confounding correlations from global fluctuations, correlation between NFA and VWFA, and noise.

**FIGURE 2:**
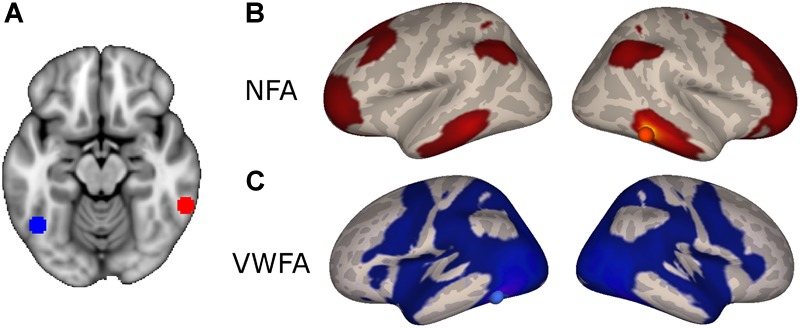
Differential connectivity. **(A)** NFA (red) and VWFA (blue) seed used in the functional connectivity analyses, overlaid onto the MNI standard template. **(B)** Regions with stronger connectivity to NFA than VWFA, red sphere represents NFA seed. **(C)** Regions with stronger connectivity to VWFA than NFA, blue sphere represents VWFA seed.

The NFA-specific network included bilateral clusters in the lateral aspect of the ITG, the IPS, the superior frontal gyrus, and in the rostrolateral prefrontal cortex (Figure 2B; *p* < 0.05, FDR corrected). No differential connectivity were found in mesial areas, medial temporal lobe or striatum. When the left NFA was used as a seed region (obtained by flipping the original seed right-to-left) the results of the differential connectivity were virtually identical (Supplementary Figure 3).

The VWFA-specific network of areas comprised of the lateral occipito-temporal cortex, the superior and medial parietal areas, motor and premotor areas and Broca’s area (pars opercularis and triangularis of the IFG and frontal operculum) and its homolog in the right hemisphere (Figure 2C).

Note that when observing the direct (as apposed to differential) voxel-wise connectivity of NFA and VWFA, these two regions were significantly connected with most of the cortex (data not shown). This is probably due to the high number of subjects included in the analysis: with 437 subjects even a marginal correlation (e.g., *r* = 0.1) is significant. This highlight the importance of observing differential correlation rather than direct one.

Supplementary Figure 1 report the direct functional connectivity of NFA and VWFA as Pearson’s correlation coefficients.

### Age-Related Development of Connectivity

In order to observe how NFA connectivity changed during development we first computed the connectivity to NFA in each voxel, and secondly how this connectivity changed with age. The corresponding analysis was then performed for VWFA.

As stated in the method, we tested a linear model, an hyperbolic model and a second order model for the association between age and connectivity. Only the first model led to significant clusters of association, thus the results of this analysis will be the focus of the rest of the manuscript.

**FIGURE 3:**
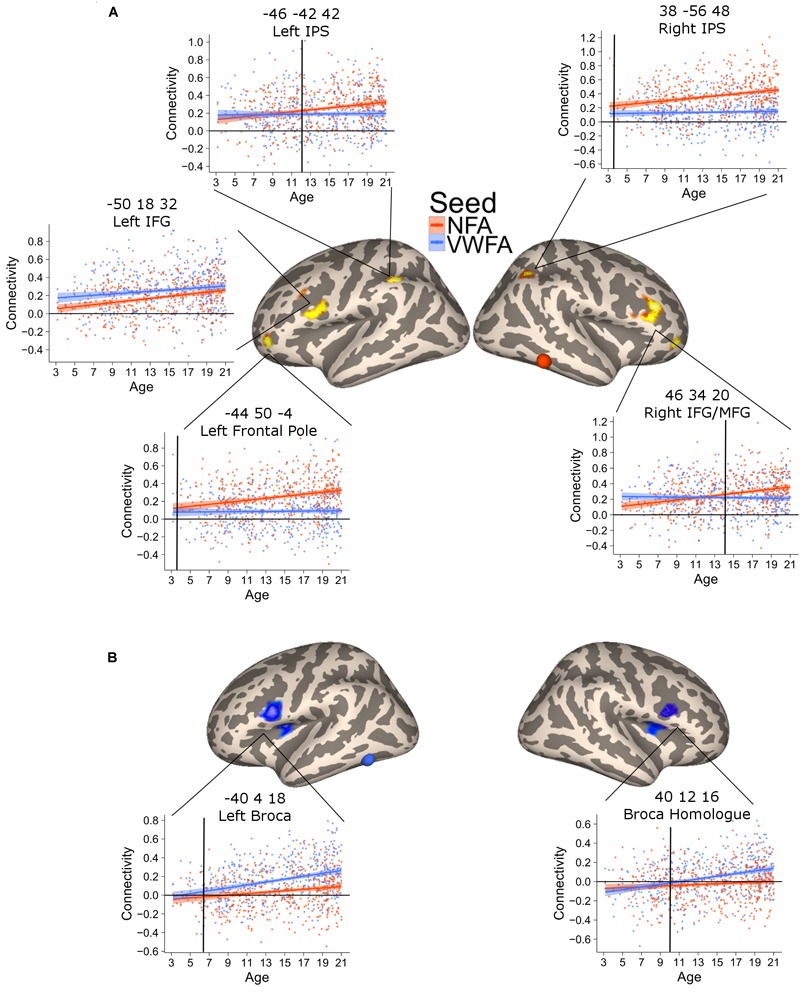
Clusters of significant association between connectivity and age for VWFA (blue) and NFA (red). The shaded area represents the 95% confidence interval of the parameter. Black vertical lines represent the age at which correlation to NFA and VWFA differ significantly. **(A)** Areas with a significant increase in connectivity to NFA with age; two bilateral clusters extending in the inferior and middle frontal gyrus [left clusters: *x* = –50, *y* = 18, *z* = 32, p(FDR) < 0.001; right cluster: *x* = 46, *y* = 34, *z* = 20, p(FDR) < 0.001], two bilateral clusters within the intraparietal cortex [left clusters: *x* = –46, *y* = 42, *z* = 42, p(FDR) = 0.04; right cluster: *x* = 38, *y* = –56, *z* = 48, p(FDR) = 0.017], a cluster in the cerebellum [*x* = 30, *y* = –66, *z* = –42, p(FDR) = 0.04] and one cluster in the left frontal pole [*x* = –44, *y* = 50, *z* = –4, p(FDR) = 0.046]. **(B)** Clusters of significant association between connectivity with VWFA and age; bilateral regions in the ventral prefrontal cortex [left clusters: *x* = –40, *y* = 4, *z* = 18, p(FDR) < 0.001; right cluster: *x* = 40, *y* = 12, *z* = 16, p(FDR) < 0.001].

This analysis revealed six clusters where NFA connectivity significantly increased with age, including bilateral regions in the intraparietal cortex, bilateral regions in the middle frontal gyrus, left rostrolateral PFC and one region in the cerebellum (not shown) [Figure 3A; all p(FDR) < 0.05]. The correlation in the parietal clusters was further characterized by superimposing these onto a cytoarchitectonic map of the horizontal part of the intraparietal cortex (hIPS) 1, 2, and 3 (Choi et al., 2006; Scheperjans et al., 2008a,b). Both clusters had their peak of correlation within the borders of the intraparietal region. The extent of the clusters was not confined to a particular cytoarchitectonic area, but covered both hIPS1 and hIPS2 (Supplementary Figure 4). Importantly, the clusters in the left IPS as well as the clusters in the left and right middle temporal gyrus partially overlapped with the network identified when contrasting the connectivity of NFA with the connectivity of VWFA (Supplementary Figure 5). Thus, these clusters show age-related increases as well as connectivity specific to NFA.

The connectivity to VWFA that increased with age included two bilateral clusters extending in the IFG (pars opercularis) and the frontal operculum (corresponding to Broca’s area and its homolog in the right hemisphere) [Figure 3B; all p(FDR) < 0.05]. These clusters overlapped with the network of areas revealed when comparing VWFA connectivity with NFA connectivity (Supplementary Figure 5). Table 2 report peak(s), cluster size and associated statistics for the results of the voxel-wise analysis in PING.

**Table 2:** Voxel-wise analysis in PING.

	Peak MNI coordinates (*x, y, z*)	Extent	T statistic
**NFA > VWFA**			
	62, -46, -16	60432	Inf
**VWFA > NFA**			
	-62, -42, -14	3485	Inf
	10, -50, 80	853	6.38
**NFA correlation with age**			
	46, 34, 20	1028	5.59
	38, -56, 48	346	4.85
	-44, 50, -4	218	4.63
	-50, 18, 32	895	4.56
	30, -66, -42	240	4.41
	-46, -42, 42	250	4.35
**VWFA correlation with age**			
	-40, 4, 18	850	6.77
	40, 12, 16	718	6.45

*This table reports the peak coordinates, the cluster extent in voxels and the associated T statistic of the clusters found for the voxel-wise analyses performed on the PING dataset.*

The previous analysis identified areas where connectivity changed with age. An additional analysis was aimed at confirming that this increase was greater for NFA and VWFA, respectively. To this end, we extracted the connectivity values between the NFA-age related clusters and VWFA, as well as the connectivity between the VWFA-age related clusters and NFA. For each cluster, we tested a mixed linear model with connectivity between the cluster and VWFA/NFA as dependent variable and seed (i.e., VWFA or NFA), age and their interaction as independent variables. We accounted for repeated measure by including in the analyses the subjects as random effect. A significant interaction between age and seed would show a difference in the slope of the association between VWFA connectivity and age, and NFA connectivity and age, thus suggesting a specificity of association. For all clusters the analyses confirmed that the association between connectivity and age was stronger for the relevant seed (all *p*s < 0.01) (Figures 3A,B), except for the left inferior and middle frontal gyrus whose increase in connectivity with age was similar for NFA and VWFA. Note that this analysis was aimed exclusively at assessing possible differences in the slope of the association with age between the connectivity with NFA and VWFA, rather than specificity. For this reason, interaction between age and region must be interpreted with caution.

The plots of connectivity and age (Figure 3) indicated that differential connectivity emerges at different time points in different regions. To determine the age at which the connectivity with NFA and with VWFA diverged (i.e., time at which the connectivity between a cluster and NFA became significantly higher than the connectivity between that cluster and VWFA, or vice versa) we subtracted the connectivity value with NFA from the connectivity value with VWFA and vice versa, for each of the clusters in Figure 3, thus creating a delta connectivity score. Next, we fitted a linear model with the previously calculated delta connectivity score as the dependent variable, and age as the independent variable. We calculated the fitted values for this model and their associated 95% confidence interval. When the confidence interval of the fitted values ceases to cross the zero line one can assume that the connectivity of the cluster at hand differs between VWFA and NFA. This age is marked with black vertical bars in Figure 3. Moreover, we repeated the above process 5,000 times bootstrapping our sample (i.e., sampling with replacement). For each cluster and each of the 5,000 repetitions we identified the age of differentiation, thus obtaining 5,000 age of differentiation for each cluster. We used these 5,000 values in order to calculate a confidence interval of the age of differentiation. This confidence interval is used as an indication of significant difference in the age of differentiation: if the confidence intervals of the age of differentiation for two ROIs do not overlap, this suggests the age of differentiation for these two ROIs is different.

This analysis verified a striking difference in the time of differentiation of the different regions in the NFA network. The right IPS showed a higher connectivity with NFA relative to VWFA since the youngest age (3.5 years, CI [3.46–3.49]), while its homolog on the left started showing a higher connectivity with NFA than with VWFA much later (11.5 years, CI [11.45–11.53]). Connectivity to the right IFG and the ventral aspect of the frontal pole became greater for NFA relative to VWFA only at 13.3 (CI [13.28–13.32]) years, while the differentiation for the left ventral aspect of the frontal pole occurred much earlier (4.8 years, CI [4.73–4.81]).

The connectivity between VWFA and the left IFG emerged at 5.8 years (CI [5.79–5.88]); the connectivity to the right IFG at 10.2 years of age CI [10.12–10.19] (Figure 3).

**FIGURE 4:**
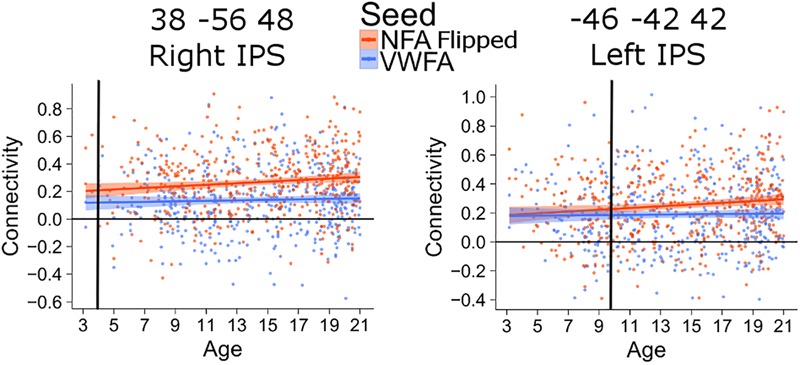
Age of differentiation for NFA flipped and VWFA. The plots depict the association between connectivity with VWFA and age (blue) and between connectivity with NFA (flipped) and age (red). The shaded area represents the 95% confidence interval of the parameter. Black vertical lines represent the age at which correlation to NFA and VWFA differ significantly.

The connectivity between the left IPS and NFA thus emerged much later than that between the right IPS and NFA. The difference in the age of differentiation seen with this analysis may be confounded by inter-hemispheric change in connectivity with age (i.e., right NFA could have higher connectivity with the right IPS early during development because they are in the same hemisphere). To exclude this confound, we also analyzed connectivity between the clusters in the left and right IPS and a NFA seed placed in the left hemisphere (using a right-to-left flipped version of the NFA seed). The difference in differentiation between left and right IPS was confirmed: for the connectivity between left NFA and right IPS, the age of differentiation was at 3.9 years (CI [3.91–3.98]), while for the connectivity between left NFA and left IPS the age of differentiation was 9.8 (CI [9.77–9.92]) years (Figure 4).

The differentiation results are based on linear modeling and interpolation. To confirm that a linear fit was the most appropriate and thus that the interpolations are acceptable, we have compared the fit of two models using as independent variable age or 1 minus the inverse of age. For all clusters, the model including age minimized the Akaike Information Criterion (AIC) relative to the model with inverse age. An exception was the cluster in the left IPS (*x* = -46, *y* = -42, *z* = 42), for which the AIC was minimized by the inverse of age. However, we visually compared the fit of the linear model and of a local regression (LOESS), that fit the data without assuming any shape of the relationship (Cleveland and Devlin, 1988). The comparison showed that, for the younger ages, the LOESS regression fitting led to smaller age values than the linear regression ones, while around the ages in which we observed the differentiation, the two models were almost identical, thus confirming the age of differentiation (Supplementary Figure 6).

### Lack of Age-Effect in Somatosensory-Motor Control Areas and Effect of Movement

**FIGURE 5:**
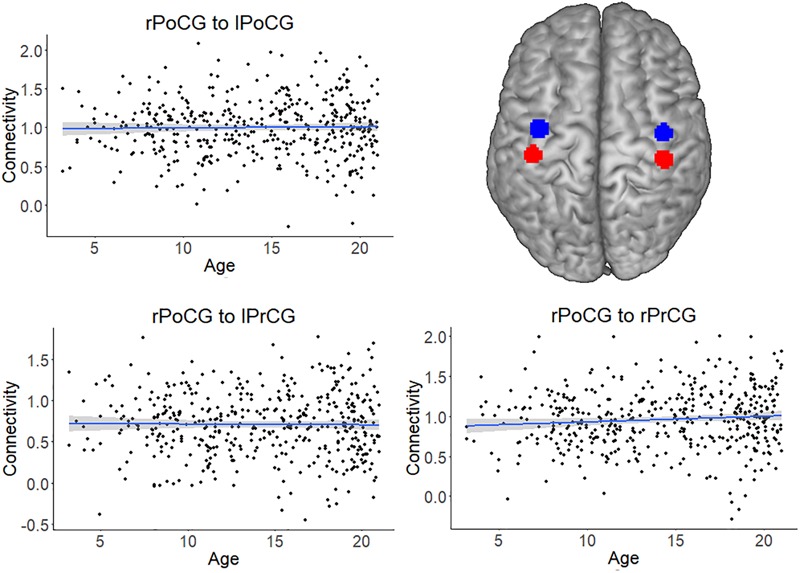
Connectivity of the somatosensory-motor network, which served as a control network. The plots depict the non-significant association between connectivity and age for the pairs right postcentral gyrus (rPoCG) – left postcentral gyrus (lPoCG), rPoCG – left precentral gyrus (lPrCG), rPoCG – right precentral gyrus (rPrCG). The shaded area represents the 95% confidence interval of the parameter. The black solid line represents the zero line. The regions of interest (precentral gyrus in red and postcentral gyrus in blue) have been overlaid on a standard template.

For all analyses, the effect of head motion was removed from the time series during preprocessing. The scatterplots and analysis of Figure 3 also shows that the increase in connectivity was specific for NFA and VWFA. To further exclude that possible age-related confounds, such as head motion or head size, explained the increase in connectivity with age, we tested the association between age and connectivity for a set of control ROIs in a somatosensory-motor network. The primary somatosensory and motor areas develop early in life. We therefore expected significant connectivity between these regions, but that this connectivity would not change with age. We selected the right postcentral gyrus (rPoCG) from the automatic anatomical labeling atlas and we calculated connectivity between this region and the left postcentral gyrus (lPoCG), as well as the right and left precentral gyrus (rPrCG, lPrCG). While the pairwise connectivity between rPoCG and the other regions was significantly different from zero (all *p* < 0.001), the association between connectivity and age was non-significant for each pair (all *p* > 0.06) (Figure 5).

As a further control analysis, we tested linear models for each cluster resulting from the age-association analysis (Figure 3, main text). For each cluster we constructed a linear model with the average connectivity between the cluster and VWFA/NFA as the dependent variable and age as well as the average framewise displacement (a compound measure of the movement occurred during the acquisition) as the independent variable, thus testing the association between connectivity and age while controlling for a global index of movement. All the clusters showed a significant association with age also when movement was included as a covariate (all *p* < 0.005).

### Functional Relevance of Connectivity Increase With Age

If the increase in connectivity in NFA and VWFA during development is related to education and training, we would expect them to be also related to improvement in mathematics and reading ability. A complete dissociation between these measures could not be expected, however, since both reading and mathematical ability are highly correlated (*r* = 0.76 in the BrainChild sample), and both are associated with chronological age and years of schooling.

To evaluate the relation to functional outcome, we turned to the third wave of a longitudinal dataset of children and adolescents (*BrainChild Dataset;* see Soderqvist et al., 2010; Dumontheil et al., 2011; Darki and Klingberg, 2015) containing 61 subjects (mean age = 16.7 ± 5.2 years, range 7.9–28.9, 33 males). Although this is a smaller dataset, it contains extensive tests of mathematics and reading which were not available for the PING dataset (see the section “Materials and Methods” for details on the measures of mathematics and reading proficiency).

After transforming both datasets to a common template, we applied the regions from the connectivity analysis in the PING dataset to the BrainChild dataset, extracted functional connectivity from each individual and related these to the behavioral measures.

**FIGURE 6:**
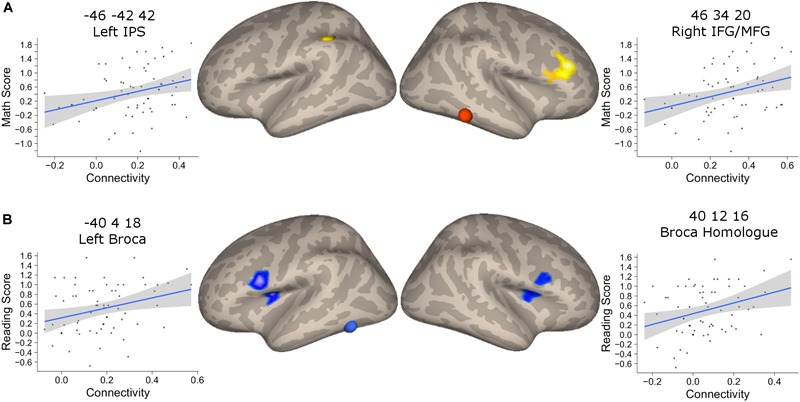
Association between connectivity and behavioral performance. **(A)** Clusters that showed a significant association between connectivity with NFA and age in the PING dataset and that showed significant association with the mathematical score in the BrainChild sample. The shaded area represents the 95% confidence interval of the parameter. **(B)** Clusters that showed a significant association between connectivity with VWFA and age in the PING dataset and that showed significant association with the reading score in the BrainChild sample. The shaded area represents the 95% confidence interval of the parameter.

We found that the mathematical performance was significantly correlated with the connectivity between NFA and the left IPS (*r* = 0.28, 95% CI [0.06, 0.52], *p* = 0.025), as well as NFA and the right IFG/MFG (*r* = 0.30, 95% CI [0.06, 0.52], *p* = 0.015) but not between NFA and the right IPS (*r* = 0.17, *p* = 0.18) (Figure 6A). None of these connectivities were significantly correlated with reading ability (all *p* > 0.07).

The connectivity between VWFA and the left and right inferior frontal regions were significantly associated with reading abilities (left cluster: *r* = 0.30, 95% CI [0.05, 0.51], *p* = 0.017; right cluster: *r* = 0.25, 95% CI [0.004, 0.47], *p* = 0.046) (Figure 6B). None of these regional connectivities showed a significant association with mathematical performance (all *p* > 0.07). Note that the statistical significances from the correlation between connectivity and behavioral indexes were corrected for multiple comparisons using FDR.

In order to show that NFA and VWFA differential connectivity was comparable in PING and BrainChild we performed the differential connectivity analysis in BrainChild. As Supplementary Figure 7 shows, NFA and VWFA differential connectivity in the BrainChild dataset was similar to the one found in PING.

## Discussion

Here, we used resting state connectivity in a large sample of children and adolescents to show the changes in connectivity of NFA and VWFA during development. The NFA-ROI showed specific connectivity, gradually increasing with age, to a network of areas located in the bilateral intraparietal cortex and dorsolateral prefrontal cortex (DLPFC), bilaterally. The increased connectivity of NFA with the left IPS and right DLPFC was also significantly related to improved mathematical abilities, but not reading. In contrast, VWFA increased its connectivity to ventral parts of the prefrontal cortex (Broca’s areas) and a homologous area in the right hemisphere. This increase in connectivity was associated with improved reading ability, but not mathematics. We did not find a strong dissociation between math and reading ability correlation within NFA and VWFA (i.e., the strength of the correlation with math and reading ability was not different within NFA and VWFA). However, these two measures are highly correlated and are both confounded by age and years of schooling. In the light of this, we believe that our results suggest some degree of specificity for NFA/math and VWFA/reading.

We chose to use a NFA ROI derived from adult literature because our aim was to observe how this region, which is specific for Arabic numerals in adults, develop its connectivity network from childhood to adulthood. Our results do not imply that NFA is category-specific in pre-school children. Moreover, it should be noted that the specificity of the NFA and VWFA ROIs has not been tested in the samples included in this paper. However, the ROI we chose as NFA was within the meta-analytical cluster found by Yeo et al. (2017) and as such can be considered representative of the NFA in the adult population.

### Developmental Change of NFA Connectivity

A surprising finding was the large difference in the age at which specific connectivity between NFA and different cortical areas emerged. The earliest development was seen for the right IPS, where differential connectivity was detected at the earliest age tested (around 3.5 years), before any formal training in mathematics. This could reflect a role of the right IPS in an early understanding of magnitude or numerosity, which is known to be present already at birth (Izard et al., 2008; Hyde and Spelke, 2011). A previous analysis of mathematical ability and white matter connectivity showed that the anterior part of the right IPS has particularly strong connections to the frontal lobe, and that the cortical thickness of this subregion is already correlated to mathematics in 6-year-old children, which was the earliest age tested (Schel and Klingberg, 2016). The fact that differential connectivity for NFA relative to VWFA was present in this region from such an early age could explain why connectivity between this region and NFA was not correlated with math achievement in the BrainChild sample: the optimal level of connectivity could have been reached early on during development and variability is no longer associated with performance beyond this level. However, the behavioral sample was older, and age-appropriate tests in younger children might have found association of performance also to the right IPS.

In contrast to the right IPS, the specific connectivity between the right NFA and the left IPS did not emerge until 11.5 years of age. This striking left-right difference was also seen when the left NFA was used as a seed, where connectivity to the right IPS was already evident at the youngest age, and connectivity to left IPS emerged at about age 10, thus excluding the interpretation that this finding was caused by an intra/inter hemispheric connectivity difference. These results are consistent with a developmental study of cortical response to numbers in children between 6 and 14 years of age (Vogel et al., 2015). In that study, both right and left IPS responded to Arabic numbers, but only for the left there was a change with age (Vogel et al., 2015). Similarly, a comparison of the left and right IPS in 4- to 9-year-old children showed that only activity in the left IPS was associated with improvement in children’s numerical discrimination acuity, and thus seemed more sensitive to education and training (Emerson and Cantlon, 2015). These reported findings are compatible with the association we found between left IPS connectivity and improvement in mathematical ability, which changes with age and education. One tentative explanation for this hemispheric difference could be that the connectivity between NFA and left IPS become more relevant when children encounter more difficult and conceptually challenging mathematical concepts in grade 5–6.

The connectivity from NFA to the right inferior and middle frontal gyrus emerged at 13.3 years and was significantly associated with mathematical ability. A meta-analysis suggested that this region is related to arithmetic processing rather than number processing (Arsalidou and Taylor, 2011). This would be consistent with the late differentiation, after years of schooling and mathematical training.

An unexpected result was the early differentiation observed for the cluster in the left frontal pole (Brodmann Area 10), although this connectivity was not significantly associated with mathematical ability. This area has been proposed to be devoted to protecting the execution of long-term plans from the immediate demand of the environment (Koechlin and Hyafil, 2007), coordination of multiple cognitive demands (Gilbert et al., 2006), and reasoning (Christoff et al., 2001; Bunge et al., 2005; Crone et al., 2009). However, we have no explanation for the early connectivity to this region. Future studies may shed light upon the role of this region in math development observing the activation and the connectivity of this region during different math tasks, and how task-related activation and connectivity change during development.

A previous study had found that the gray matter volume of a region in the left, rather than the right, fusiform gyrus predicted math gain in young children (Evans et al., 2015). Moreover, the same study found that intrinsic connectivity in the network of regions functionally connected with the cluster in the left fusiform gyrus could predict math gain. Although, at first pass, these results might seem in opposition to each other, there are at least two reasons that may explain the difference between our results and those of Evans et al. (2015). First, their seed region for connectivity analyses was identified based on an associate between gray matter volume and math gains, while our region used the previous literature as a starting point. Second, the sample included in the study of Evans et al. (2015) had a quite narrow age-range, while we use a wide age range in order to follow the development of NFA-related network.

### Developmental Change of VWFA Connectivity

The VWFA showed a differential connectivity to Broca’s area that emerged at 6 years of age, when formal reading training normally begins. The strength of connectivity was also significantly associated with reading ability, as was the connectivity to the homologous area in the right hemisphere. It is interesting to note that although the tuning of VWFA to words has been shown to stabilize around 12 years of age (Ben-Shachar et al., 2011), here we show that the integration of the VWFA with other language related areas (as Broca’s region) continue well beyond that age, linearly increasing until 20 years old (the oldest age in our sample). Although the VWFA was connected to language areas in the superior temporal gyrus and inferior parietal lobe (Figure 2) there was not the expected change in connectivity with age to these regions. One possible explanation for the lack of developmental change could be that this connectivity emerges earlier than the age range studied here, concomitant with the development of learning of the spoken language. The second, alternative, explanation is that this connectivity is hard-wired and does not change with development (Dehaene-Lambertz et al., 2006; Perani et al., 2011; Saygin et al., 2016). Although it is important not to over interpret a negative finding, the statistical power that a large sample as the one used in this study grants us suggest that this can be a true negative. The functional connectivity results we found for VWFA are somewhat in contrast prior findings of low or null functional connectivity between VWFA and reading-related regions, finding instead functional connectivity between VWFA and regions of the dorsal attentional network (Vogel et al., 2015). There are several reasons that could explain the contradiction between our results and those of Vogel et al. (2015): first of all, they used a children and adult samples composed of 22 and 23 subjects, respectively. We used a sample composed of about 400 subjects, that certainly led to an higher statistical power relative to Vogel et al. (2015). A part from statistical power issue, other analytical choices can be responsible for the differences in our results: Vogel et al. (2015) focused on direct connectivity, while we chose to focus on differential fucntional connctivity (i.e., for each voxel we looked at the difference in conncetivity with VWFA and NFA). Looking at the results of VWFA direct connectivity (Supplementary Figure 1) one can see that we indeed observed a connectivity between VWFA and some of the dorsal attentional regions found by Vogel et al. (2015). However, when compared to NFA connectivity, we observed that VWFA was *more connected than NFA* to reading-related regions. Moreover, we used a smaller smoothing kernel and smaller ROIs relative to Vogel et al. (2015): this could have led to a greater specificty in our results (i.e., the connectivity of VWFA be less influneced by its neighbors).

### Region With a Similar Development for NFA and VWFA

Interestingly, a cluster in the left IFG and MFG (*x* = -50, *y* = 18, *z* = 32) showed a similar strengthening of connectivity with age for both NFA and VWFA. This region is close to the cluster identified as Broca’s region in the analysis of the association between connectivity and age for VWFA. This cluster is also close to one of the clusters identified in a meta-analysis (Arsalidou and Taylor, 2011) as being related to arithmetic processing (*x* = -45, *y* = 30, *z* = 29). This area is less activated in children with mathematical learning disabilities during a multiplication task (Berteletti et al., 2014). The similar strengthening of connectivity of this region with both VWFA and NFA with development is in line with the idea that this region could be an interface between the verbal and the mathematical networks. It has been suggested that development dyscalculia is related to abnormal functional connectivity in the mathematical network (Rosenberg-Lee et al., 2015); our results show how the mathematical network develops in healthy children and can inform future study investigating the timing of this development in children with learning disabilities or dyscalculia.

### Mechanisms of NFA and VWFA Specificity

A key question is how VWFA and NFA networks develop. One hypothesis is that there is originally a wide-spread connectivity and that specialization emerges by competition: i.e., that both VWFA and NFA are anatomically connected to Broca’s area, but that VWFA-Broca’s connections gradually are strengthened and NFA-Broca’s connections are lost. In Figure 3, the connectivity of NFA and VWFA are directly compared. In the areas where connectivity to NFA increased with age there was no simultaneous decrease in connectivity to VWFA, and vice versa. This suggests that there is no competition in connectivity between NFA and VWFA. Instead, the anatomical connections between these areas seem to be present at an early stage, and then are strengthened, tentatively by activity-dependent synaptic plasticity. This is also consistent with the hypothesis of biased connectivity put forward by Hannagan et al. (2015).

## Conclusion

In conclusion, we have shown that parts of the mathematical core-network connected to NFA develop at a very early age, possibly reflecting an inborn connectivity. Other parts of the network take years to form through development and education. This development is also related to the gradual improvement in mathematical proficiency.

## Ethics Statement

This study was carried out in accordance with the recommendations of the Stockholm County ethics committee, with written informed consent from all subjects. All subjects gave written informed consent in accordance with the Declaration of Helsinki. The protocol was approved by the Stockholm County ethics committee.

## Author Contributions

FN conceived the study, planned and performed the statistical analyses, and wrote the first draft of the paper. MS performed the statistical analyses and participated in writing and revising of the paper. TK conceived the study, directed data acquisition, and participated in writing and revising the manuscript. The PING consortium provided part of the data.

## Conflict of Interest Statement

The authors declare that the research was conducted in the absence of any commercial or financial relationships that could be construed as a potential conflict of interest.

## Footnotes

1 http://ping.chd.ucsd.edu/

2 http://pingstudy.ucsd.edu/welcome.html

3 http://www.nitrc.org/projects/conn

4 http://www.nitrc.org/projects/artifact_detect/
