# Supplementary material for: Connectivity of the Human Number Form Area Reveals Development of a Cortical Network for Mathematics
Source: Front Hum Neurosci. 2018 Nov 26;12:465. doi: 10.3389/fnhum.2018.00465 (PMC6275176; doi:10.3389/fnhum.2018.00465)
Supplement: Supplementary file 1 [file Data_Sheet_1.docx]

***LEGENDS***

***
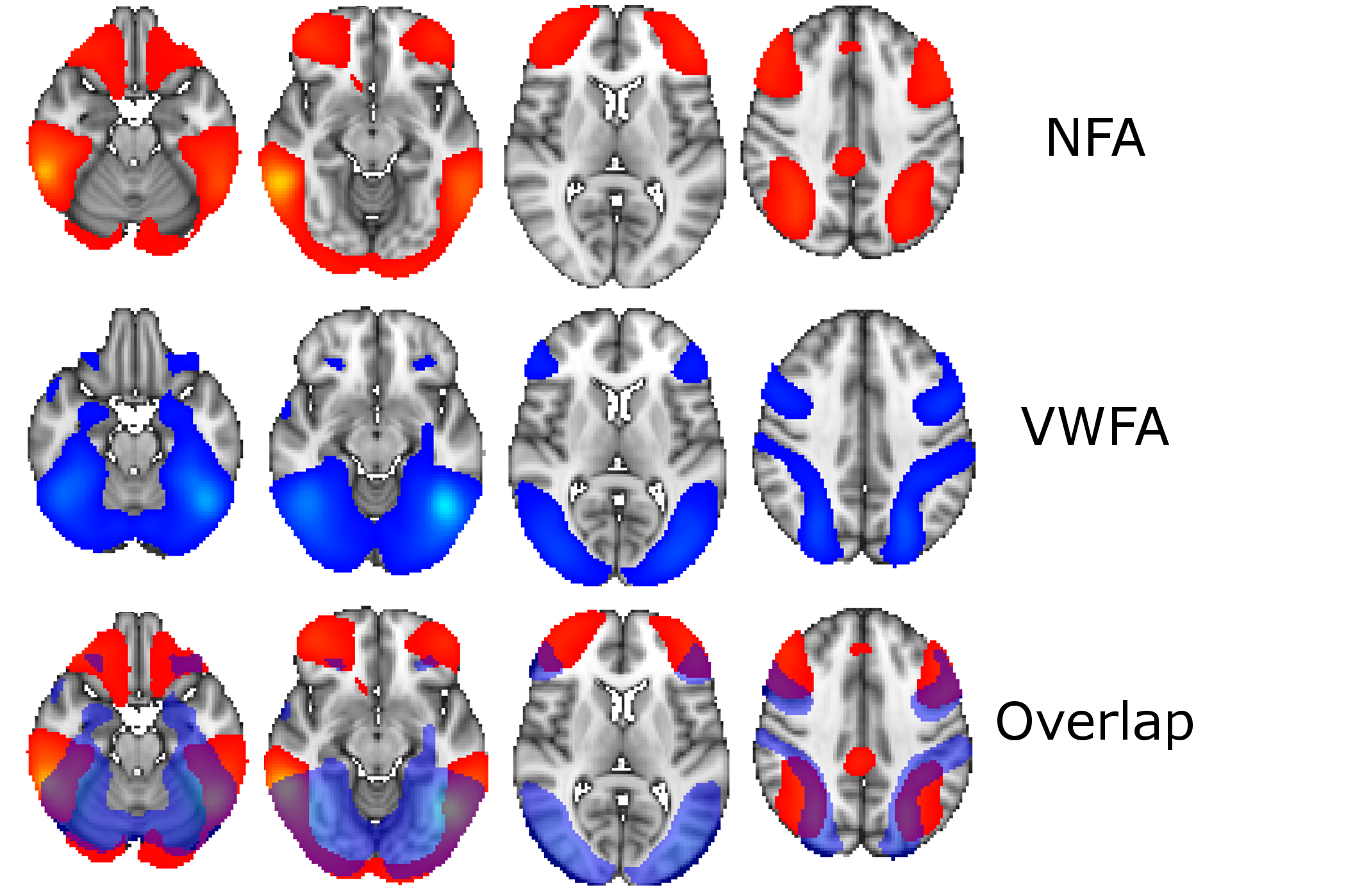
***

*Supplementary Figure 1. Direct functional connectivity of NFA and VFWA and their overlap. This figure shows the direct functional connectivity of NFA (upper panel) and VWFA (middle panel) as Pearson’s correlation coefficient thresholded at .1. The lower panel present the two networks overlapped.*

***
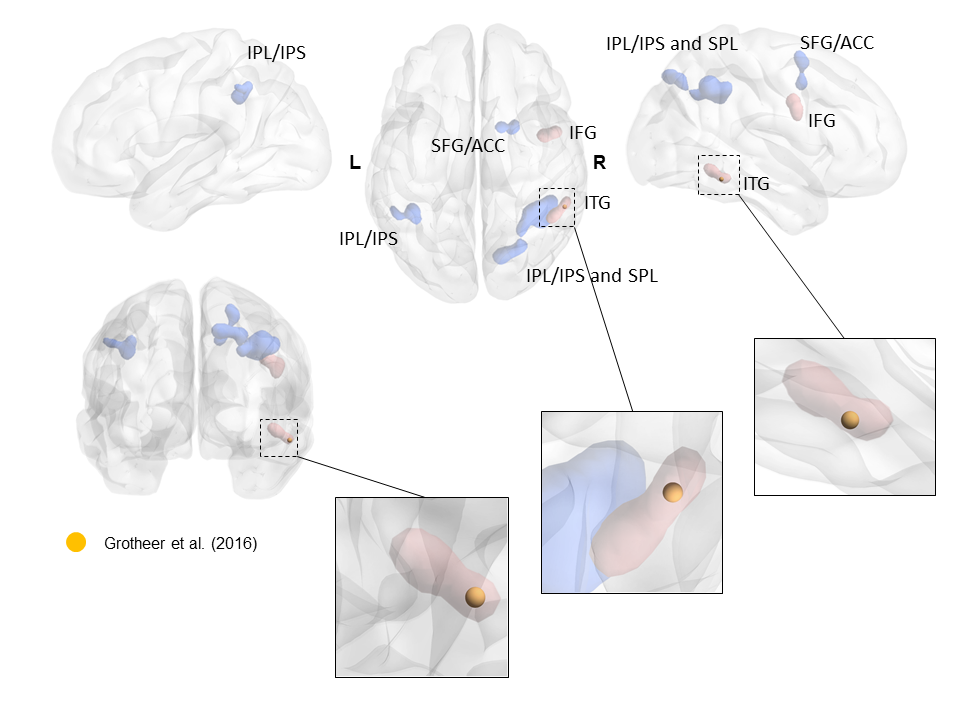
***

*Supplementary Figure 2. Overlap of the NFA-ROI (peak from Grotheer et al., 2016b) with the meta-analytical cluster in the IFG found by Yeo et al.(2017). The red transparent cluster in the inset is the NFA as found in the study by Yeo and colleagues (2017), while the yellow sphere in the inset is the center of gravity of the cluster found by Grotheer and colleagues (2016b)This figure is courtesy of Darren Yeo and Gavin Price.*


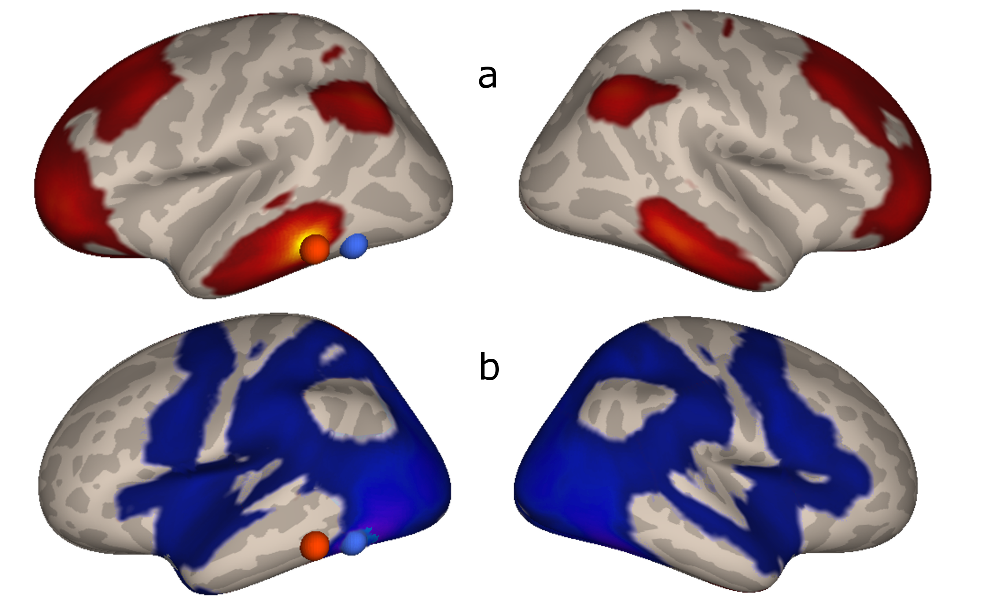


*Supplementary Figure 3. Differential connectivity of NFA (upper panel) and VWFA (lower panel) when using a right-to-left flipped version of the NFA seed in the PING dataset.*

*
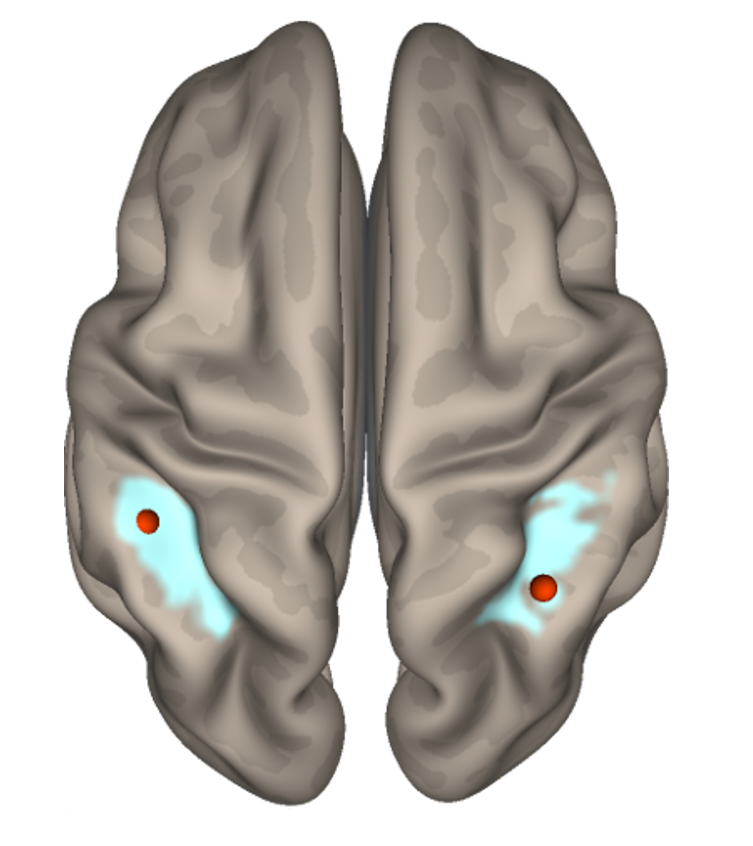
*

*Supplementary Figure 4. Overlap of IPS-cluster identified by the differential connectivity of NFA (i.e. the contrast NFA > VWFA) and the left and right IPS as defined cytoarchitectonically in Choi et al., 2006; Scheperjans et al., 2008b; Scheperjans et al., 2008a. hIP1, hIP2 and hIP3 have been merged into one region and represented in pale blue. The sphere represents the peak of functionally connectivity with NFA we found in the left and right IPS.*

*
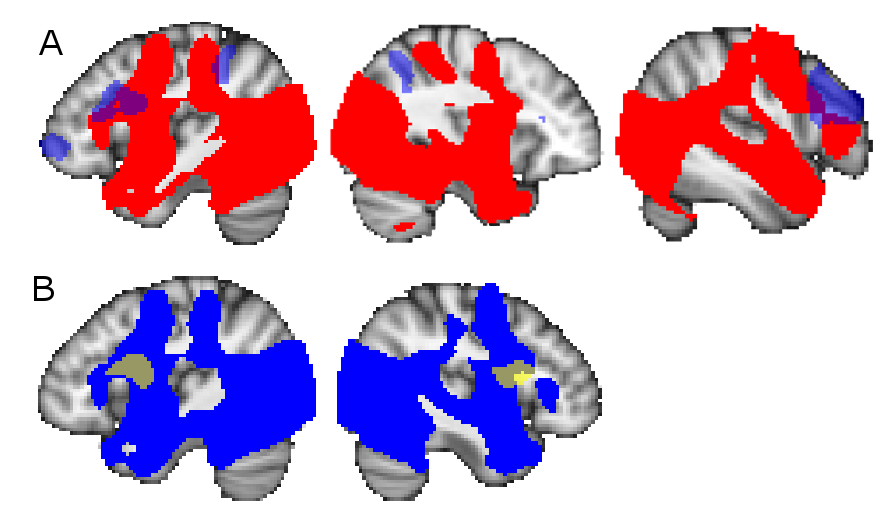
*

*Supplementary Figure 5. Overlap of the differential connectivity and correlation with age. Panel A shows the overlap of the differential connectivity of NFA (contrast NFA > VWFA) in red, and the clusters of correlation between NFA connectivity and age (in blue). The overlap is shown in purple. Panel B shows the overlap of the differential connectivity of WFA (contrast VWFA > NFA) in blue, and the clusters of correlation between VWFA connectivity and age (in yellow). The overlap is shown in green.*


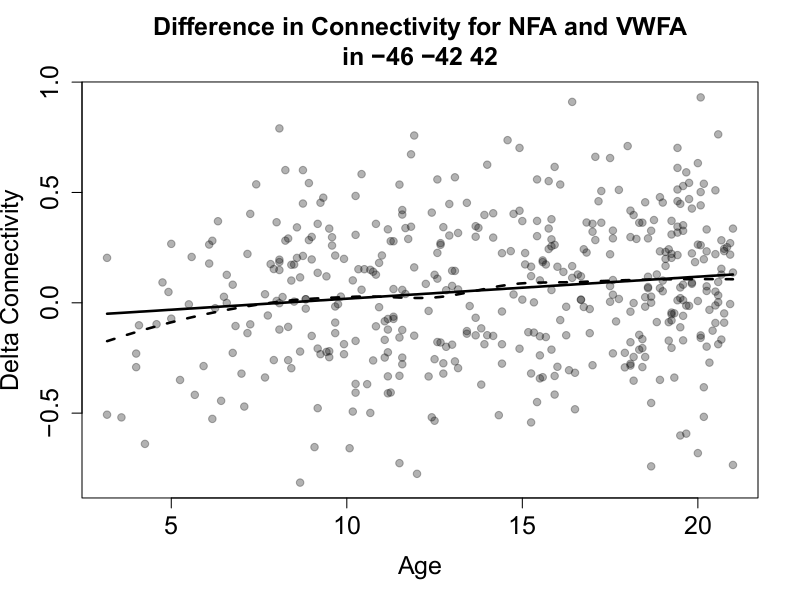


*Supplementary Figure 6. Comparison of linear and loess fit for the cluster in -46 -42 42. This was the only clusters in the analyses of the age of differentiation for which the AIC was minimized by 1 – 1/age rather than linear age. The plot shows that for a loess (non parametric) fit of the data (dashed line) the predicted delta connectivity between the two seed is lower than for the linear fit (solid line).*


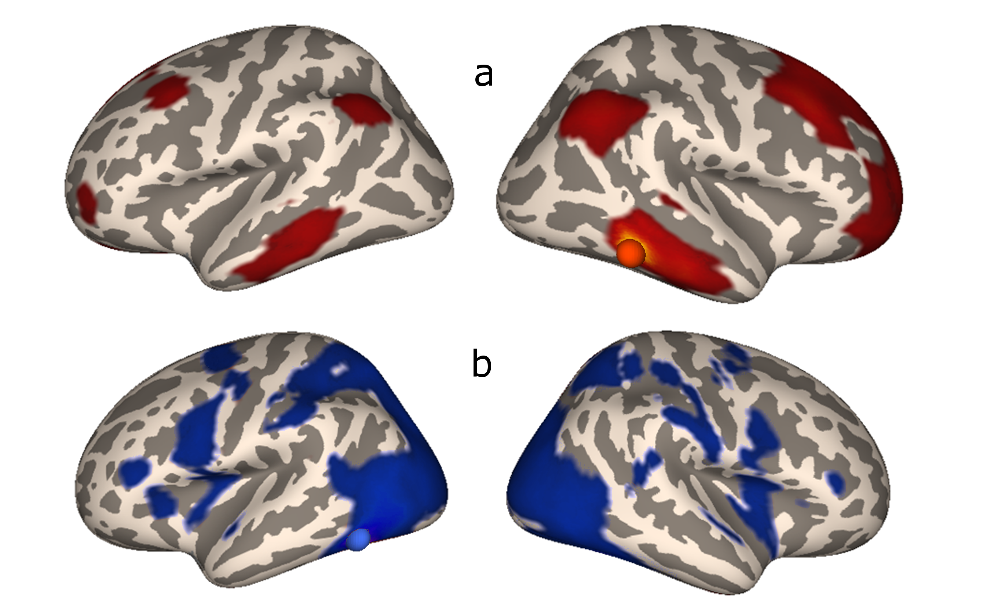


*Supplementary Figure 7. Differential connectivity for NFA (upper panel) and VWFA (lower panel) in the BrainChild sample.*

*Rasch model*

The mathematical score as well as the reading score were transformed in ability scores using the Rasch model. The Rasch model posit that the probability that a specific subject passes a specific item is function of two parameters: the item difficulty (δi) and the subject ability (θn). In this case, if we write Xni to denote the binary outcome, where 1 = “pass,” 0 = “fail” then the probability (Pr) of passing item i is written: Pr[Xni=1]= exp(θn - δi )/(1 + exp(θn - δi)). When using age-specific test, as we did in this study, calculating the ability score for each age-range lead to an index that is comparable across ages.
